# The Empathizing-Systemizing Theory, Social Abilities, and Mathematical Achievement in Children

**DOI:** 10.1038/srep23011

**Published:** 2016-03-14

**Authors:** Emily Escovar, Miriam Rosenberg-Lee, Lucina Q. Uddin, Vinod Menon

**Affiliations:** 1 Department of Psychiatry and Behavioral Sciences Stanford University, Stanford, CA USA; 2 Department of Psychology, University of Miami, Coral Gables, FL USA

## Abstract

The Empathizing-Systemizing (E-S) theory describes a profile of traits that have been linked to autism spectrum disorders, and are thought to encompass a continuum that includes typically developing (TD) individuals. Although systemizing is hypothesized to be related to mathematical abilities, empirical support for this relationship is lacking. We examine the link between empathizing and systemizing tendencies and mathematical achievement in 112 TD children (57 girls) to elucidate how socio-cognitive constructs influence early development of mathematical skills. Assessment of mathematical achievement included standardized tests designed to examine calculation skills and conceptual mathematical reasoning. Empathizing and systemizing were assessed using the Combined Empathy Quotient-Child (EQ-C) and Systemizing Quotient-Child (SQ-C). Contrary to our hypothesis, we found that mathematical achievement was not related to systemizing or the discrepancy between systemizing and empathizing. Surprisingly, children with higher empathy demonstrated lower calculation skills. Further analysis using the Social Responsiveness Scale (SRS) revealed that the relationship between EQ-C and mathematical achievement was mediated by social ability rather than autistic behaviors. Finally, social awareness was found to play a differential role in mediating the relationship between EQ-C and mathematical achievement in girls. These results identify empathy, and social skills more generally, as previously unknown predictors of mathematical achievement.

Early elementary school is a period of rapid development in mathematical skills and children show significant individual differences in their abilities in this domain. There is growing interest in identifying not just the cognitive, but also the social factors that contribute to such differences^1^,^2^,^3^,^4^. Baron-Cohen and colleagues have proposed a theory that situates mathematical ability within a general socio-cognitive framework with two underlying mental processes: empathizing and systemizing^5^,^6^. Empathizing is the drive to identify another person’s perspective and emotions and generate an appropriate socio-emotional response, while systemizing is the tendency to analyze and explore a system and extract underlying rules that govern its behavior^5^. While mathematics is purported to be an example of an ability requiring systemizing^6^, there has been little empirical support for a direct link between the socio-cognitive constructs outlined by the Empathizing-Systemizing (E-S) theory and math achievement in children. Here for the first time we investigate the relationship between empathizing, systemizing, and math achievement in typically developing (TD) children.

## The Empathizing-Systemizing Theory in ASD

The E-S theory arose out of an examination of core features of autism spectrum disorder (ASD)^5^. ASD is characterized by deficits in social development, delays or lack of development in communication, and unusually repetitive behaviors and narrow interests^7^. Baron-Cohen^5^ posited that the core features of autism can be explained as individual variation on the dimensions of empathizing and systemizing. According to the theory, social and communication deficits can be explained as a low tendency to empathize^8^,^9^, while narrow interests, repetitive actions and specialized skills can be explained as a high tendency to systemize^10^,^11^.

The E-S theory has been extended to neurotypical individuals. The extended theory, sometimes framed as the Extreme Male Brain theory^5^, posits that ASD represents an exaggeration of gender differences in the TD population. Support for this proposal comes from findings of sex differences in empathizing and systemizing in TD adults^6^,^12^. Adult females tend to score higher in empathizing, as measured by the Empathy Quotient (EQ) while adult males score higher in systemizing, as measured by the Systemizing Quotient (SQ)^6^,^12^. This pattern of gender differences was replicated by Auyeung and colleagues^13^ in children (ages 4 to 11 years). Previous research also tested for sex differences in the discrepancy between empathizing and systemizing scores (computed as a ‘Difference score’). In both children and adults, males tend to have higher Difference scores, meaning they are relatively higher in systemizing and lower in empathizing than their female counterparts^12^,^13^. Difference scores can also be used to categorize individuals into ‘brain types.’ Type S individuals have greater systemizing abilities relative to empathizing abilities, while Type E individuals have greater empathizing abilities relative to systemizing abilities. Finally, Type B individuals display a balanced profile between the two abilities^12^,^13^. Extreme categories were also assigned for individuals that showed significant discrepancies (top and bottom 2.5 percentile on the Difference score). These brain types have been cited as useful in describing sex-typical behavior, with males tending to be categorized as Type S and females as Type E^12^.

## Systemizing and Mathematics

Much of the existing work on the E-S theory cites mathematics as a key domain of systemizing skills, based on links between heightened mathematical ability and superior systemizing abilities in college students and adults with ASD^6^,^14^,^15^. Mathematics is a rule-based system, so it is a logical step to hypothesize enhanced math achievement stems from enhanced systemizing abilities. Mathematics undergraduate majors are more likely to have a diagnosis of ASD or have an immediate family member diagnosed with the disorder^14^. Also, it has been reported that students in the natural sciences (including engineering and mathematics) have more relatives with ASD than students in the humanities^16^. In addition, students majoring in mathematics score higher than students majoring in the humanities or social sciences on the Autism Quotient questionnaire^17^. Despite these intriguing findings, there is surprisingly little direct evidence for a link between either systemizing or Type S (greater discrepancy between systemizing and empathy quotients) and mathematics ability in either individuals with ASD or neurotypical individuals.

Only one study to date has tested for a direct link between systemizing and math achievement. Morsanyi and colleagues^18^ assessed math achievement and systemizing in 93 adult female participants. They found no relationship between systemizing and math performance; however, they did find a relation between math performance and attitudes toward statistics, statistics-related anxiety, and level of confidence in mathematical problem solving^18^. In a second study with 125 adults (70 female), they found that systemizing abilities are correlated with performance on a mechanical reasoning test and with scores on a self-report measure of spatial thinking styles^18^, but math achievement was not assessed. The dearth of empirical research in this area calls for a comprehensive battery of math achievement to better characterize the differential relations between systemizing and various components of math ability.

## Empathizing, Gender, and Mathematics in Children

No studies to date have explicitly examined the relationship between empathizing and math achievement; however, research on the social transmission of attitudes toward math, and the effect these attitudes may have on children’s math learning, may have some bearing on this question. Previous research has found that attitudes toward math, math anxiety, endorsement of math-related gender stereotypes, and expectations of failure in math are often socially transmitted and can influence math achievement and choices to pursue science and math related careers^2^,^4^,^19^,^20^,^21^. Furthermore, the transmission of negative attitudes toward math is gender specific^22^. Beilock and colleagues^1^ found that at the beginning of the school year, teachers’ math anxiety was unrelated to their students’ math performance; however, by the end of the year, the teachers’ math anxiety predicted the likelihood that girls, not boys, would hold stereotypic beliefs regarding the poor performance of girls in math. Moreover, the teachers’ math anxiety also predicted the girls’ math achievement, suggesting that transmission of these attitudes has important consequences for academic performance^1^. Both math anxiety and, in females, stereotype threat (awareness of negative stereotypes about how one’s social group should perform) have been linked to situational difficulties in math performance driven by intrusive thoughts occupying cognitive resources otherwise dedicated to math processing^2^,^23^. Thus, a secondary aim of the present study was to examine empathizing in relation to math achievement and relevant affective and social constructs such as math anxiety, social responsiveness, and their potential interactions with gender.

Research on gender differences in math achievement has mainly focused on middle and high school populations. This is, in part, due to the fact that there is little evidence of significant gender differences in math performance in elementary school^22^,^24^,^25^. There is, however, evidence of an earlier emergence of gender differences in precursors of math achievement, including spatial ability^26^,^27^, strategy use^28^, and math anxiety^2^. Aunola and colleagues^29^ also found that rates of growth in performance were faster for boys than for girls between preschool and 2^nd^ grade. Although there is little evidence for gender differences in math achievement among elementary school children, differential predictors of performance in this age range could represent previously unknown sources of divergent trajectories in math skills for boys and girls.

## The Relationship between Empathizing-Systemizing and Mathematical Achievement in Children

The aim of the present study was to investigate the relationship between E-S measures and math achievement in TD children. We collected standardized measures of math achievement in children indexing two distinct components of mathematics: a pure measure of symbolic calculation skills and a contextualized measure of mathematical problem solving, in a large sample of 112 children (57 girls) aged 7 to 12. Parents of the participants completed the EQ-C/SQ-C, a measure of empathizing and systemizing in children. We controlled for known predictors of math achievement with measures of intelligence and reading achievement.

Following Baron-Cohen’s proposal^5^, we predicted that systemizing would be positively correlated with math achievement. In addition, we predicted that Type S individuals would exhibit greater math achievement relative to Type E individuals. Given the male advantage in systemizing in children^13^, a link between systemizing and math achievement at an early age would suggest that systemizing tendencies may contribute to gender differences in math achievement. We further expected that girls would have higher empathizing scores than boys. While the paucity of research on this topic precluded a strong *a priori* prediction for a relationship between empathizing and math achievement, we tested the hypothesis that the ability to take another’s perspective may represent a conduit for the transmission of harmful and stereotyped attitudes towards math, potentially suggesting a negative relationship between empathizing and math achievement, especially in girls. In order to investigate the relationship between math achievement and social skills more generally, we used the Social Responsiveness Scale^30^. Based on known gender differences in empathizing and systemizing, we conducted our analyses in the entire group first and then separately in boys and girls.

## Results

### SQ-C and EQ-C are Uncorrelated

In line with the TD sample in the study conducted by Auyeung and colleagues^13^, there was no relation between EQ-C and SQ-C scores. This suggests the constructs of empathizing and systemizing are independent of one another (*r*(110) = 0.086, *p* = 0.37).

### SQ-C and EQ-C Differ by Gender

Boys and girls did not differ in age, the Wechsler Abbreviated Scale of Intelligence^31^ full-scale IQ (FSIQ), reading achievement as measured by the Woodcock Johnson III (WJ-III)^32^ Basic Reading composite, or math achievement as measured by WJ-III mathematics subscales (Table 1). There was a marginally significant gender difference on SQ-C, with boys scoring higher than girls. In contrast, boys and girls differed significantly on EQ-C with girls scoring higher than boys (Table 1).

### Relation between SQ-C and Mathematical Achievement

Pearson correlations were used to examine the relationship between systemizing and two distinct components of math achievement – symbolic arithmetic ability as measured by the WJ-III Calculation Skills composite, and mathematical reasoning skills, as measured by the WJ-III Applied Problems subtest. Table 2 summarizes the relation between SQ-C, and the cognitive measures. SQ-C was not correlated with the Calculation Skills composite (*r(*110) = 0.07, *p* = 0.44), however, it was marginally positively correlated with the Applied Problems subtest (*r*(110) = 0.16, *p* = 0.08). Given the marginally significant gender differences in SQ-C scores, we then examined these relations in each gender. The relationships between SQ-C and the math measures were not significant in separate groups of boys or girls (Table 2).

Since Applied Problems was strongly correlated with FSIQ and the Basic Reading composite (Supplementary Table S1; *r*(110) = 0.64, *p* < 0.001 and *r*(110) = 0.57, *p* < 0.001 respectively), we further examined the relationship between SQ-C and Applied Problems after accounting for FSIQ and reading achievement using a hierarchical regression analysis. The domain general cognitive capacities of FSIQ and the Basic Reading composite scores accounted for 50.1% (*Adjusted R*^*2*^ = 0.49) of the variance in Applied Problems scores (*F*(2, 109) = 54.69, *p* < 0.001). Adding SQ-C to the model did not account for significant additional variance (*F change*(1, 108) = 1.45, *p* = 0.23; Supplementary Table S2). Taken together, these results suggest that SQ-C is not an independent predictor of math achievement in children.

### Relation between EQ-C and Mathematical Achievement

Table 3 summarizes the relation between EQ-C and cognitive measures. There was a negative correlation between EQ-C and the Calculation Skills composite score with greater empathizing predicting lower math achievement (*r*(110) = −0.22, *p* = 0.02). EQ-C was not significantly correlated with Applied Problems (*r*(110) = −0.01, *p* = 0.91). To further examine the relationship between EQ-C and the Calculation Skills composite we conducted a hierarchical regression analysis after accounting for FSIQ and the Basic Reading composite. The domain general predictors accounted for 23.4% (*Adjusted R*^*2*^ = 0.22) of the variance (*F*(2, 109) = 16.61, *p* < 0.001). Including EQ-C in the model accounted for an additional 3.8% of the variance in math achievement (*F change*(1, 108) = 5.71, *p* = 0.02) (Table 4). These results demonstrate that empathizing is a previously unknown predictor of math skills in TD children.

### The Relationship between EQ-C and Mathematical Achievement in Boys vs. Girls

**Figure 1:**
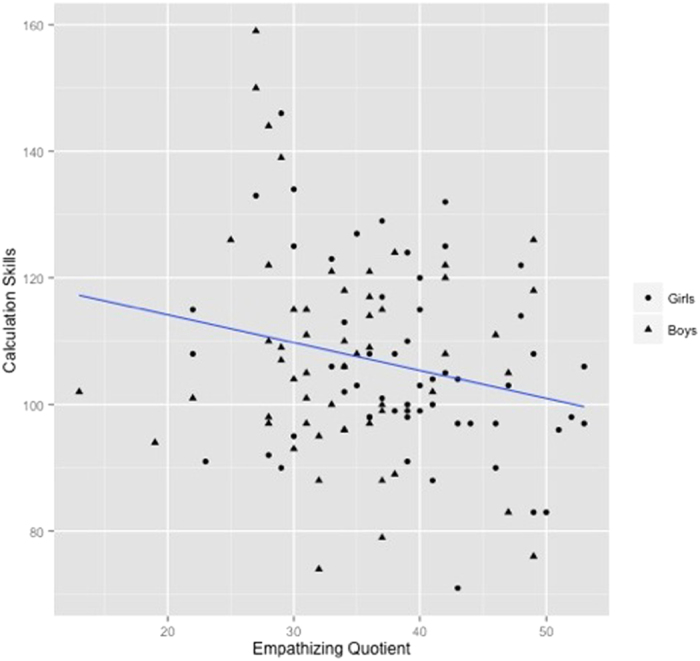
Relation between EQ-C and Math Calculation Skills (r = −0.22, p < 0.05).

Given the significant gender differences in EQ-C scores, the relation between EQ-C and the Calculation Skills composite was then examined separately in boys and girls. The relationship was significant in girls (*r*(55) = −0.29, *p* = 0.03), but not in boys (*r*(53) = −0.14, *p* = 0.32; Fig. 1); however, these effects were not statistically different from each other (*z* = −0.81, *p* = 0.42). Regression analyses, conducted separately in each group, revealed that the variance accounted for in the Calculation Skills composite by EQ-C, after accounting for FSIQ and the Basic Reading composite was significant in girls (*R*^*2*^ *change* = 0.07, *p* = 0.02), but not in boys (*R*^*2*^ *change* = 0.02, *p* = 0.25). Finally, when gender was entered into the model as a fixed factor in the full group model, EQ-C did not significantly interact with gender (*F*(1, 106) = 0.55, *p* = 0.46). These results demonstrate that gender is not a significant predictor of the relation between empathizing and math achievement.

### EQ-C/SQ-C Discrepancy and Mathematical Achievement

We also examined the discrepancy, or difference, between systemizing and empathizing as it relates to math achievement. In line with Auyeung and colleagues^13^, the genders differed on the discrepancy between SQ-C and EQ-C scores (*t*(110) = −4.11, *p* < 0.001), with boys having on average positive scores (*M* = 0.04, *SD* = 0.09) and girls having on average negative scores (*M* = −.03, *SD* = .09).

We next examined the relationship between the math measures and the Difference score. The Difference score was positively correlated with the Calculation Skills composite (*r*(110) = .22, *p* = .02), but was not correlated with Applied Problems (*r*(110) = .13, *p* = .18). In order to test whether the Difference score, per se, rather than just the known empathizing effect was driving the relationship between the Difference score and the Calculation Skills composite, we ran a stepwise regression analysis. The stepwise regression tested for the unique contributions of EQ-C and the Difference score to the explained variance in the Calculation Skills composite, after accounting for FSIQ and the Basic Reading composite. The step-wise regression analysis selected EQ-C as the stronger predictor of the Calculation Skills composite, after accounting for FSIQ and the Basic Reading composite (*F change*(1, 108) = 5.71, *p* = 0.02). After adding EQ-C to the model, the Difference score was not a significant predictor and was not included in the model (*p* = 0.51).

Finally, we examined whether there were any differences in math achievement between the ‘brain type’ groups. The brain types did not significantly differ from one another on the Calculation Skills composite (*F*(2, 109) = 1.59, *p* = 0.21, *h*^*2*^ = 0.02) or Applied Problems (*F*(2, 109) = 1.89, *p* = 0.16, *h*^*2*^ = 0.03). The brain types also did not differ from one another on the Calculation Skills composite (*F*(2, 107) = 1.06, *p* = 0.35, *partial h*^*2*^ = 0.03) or Applied Problems (*F*(2, 107) = 0.41, *p* = 0.67, *partial h*^*2*^ = 0.008) after accounting for FSIQ and reading achievement.

### The Effect of Math Anxiety on the Relationship between EQ-C and Mathematical Achievement

We next sought to determine whether the negative relationship between math calculation skills and empathizing could be explained by children’s math anxiety, a known negative contributor to math achievement^2^,^4^. We used partial correlations to examine the relation between the Scale for Early Mathematics Anxiety (SEMA)^4^ and both EQ-C and the Calculation Skills composite, after accounting for FSIQ and the Basic Reading composite. SEMA scores significantly, negatively correlated with the Calculation Skills composite (*r*(110) = −0.37, *p* < 0.001); however, EQ-C was not significantly related to SEMA (*r*(110) = −0.12, *p* = 0.23). There were no differences between the genders in SEMA scores (*t*(109) = 4.50, *p* = 0.65). Thus, children’s self-ratings of math anxiety did not explain the relationship between empathizing and math achievement.

### Relationship between Social Skills and Mathematical Achievement

We then investigated whether the relationship between math achievement and empathizing could be explained by broader social abilities assessed with the Social Responsiveness Scale (SRS). The SRS has four social subscales including Social Awareness, Social Cognition, Social Communication, and Social Motivation. We also included the Autistic Mannerisms SRS subscale to assess whether the relationship between empathizing and math achievement is related to social ability, rather than an indirect connection between low empathizing and autistic behavioral tendencies. Higher scores on the SRS subscales indicate a lack of social abilities and more severe autistic mannerisms.

The Social Awareness, Social Cognition, and Social Communication subscales were negatively correlated with EQ-C (Table 5), indicating that those children with higher empathizing scores also had lower scores on the social subscales (i.e. fewer difficulties with social abilities). With the exception of the Social Motivation subscale, all the social SRS subscales were positively related to the Calculation Skills composite score, indicating that children with higher social abilities tended to have lower math skills. The Autistic Mannerisms subscale was negatively related to EQ-C; however, it was not significantly related to math achievement after accounting for FSIQ and reading achievement. These results indicate that multiple measures of social skills are related to math achievement.

### Relationship between Social Skills and Mathematical Achievement in Boys vs. Girls

We examined whether the relation between the SRS subscales, EQ-C, and math achievement differed by gender. All of the SRS social subscales were correlated with EQ-C in both boys and girls, with the exception of Social Motivation in girls; however, only in girls did SRS measures – specifically Social Awareness, Social Cognition, Social Communication, and Autistic Mannerisms – also correlate with the Calculation Skills composite (Table 5). Direct statistical comparison between the genders revealed that differences in correlation coefficients were significant only for Social Awareness (*z* = 2.18, *p* = 0.03). Thus, the relationship between social abilities and math achievement is, to a limited extent, stronger in girls than in boys.

### Social Ability as a Potential Mediator between Empathizing and Mathematical Achievement

To further characterize the role of social abilities in the relationship between empathizing and math achievement we conducted a series of mediation analyses. Since the Social Awareness, Social Cognition, Social Communication, and Autistic Mannerisms subscales were significantly related to both EQ-C and the Calculation Skills composite, either in the whole group or in girls, they were each examined in mediation analyses. First, we assessed the strength of the relationship between EQ-C and the Calculation Skills composite after accounting for FSIQ and reading achievement (*c*). Then, in separate analyses, we added each of the SRS social subscales (Awareness, Cognition, and Communication) and the Autistic Mannerisms subscale to the model and measured the remaining relationship (*c’*) and the change in strength (*ab*).

The Social Cognition subscale significantly mediated the relationship between EQ-C and the Calculation Skills composite score. Social Awareness was a marginal mediator (Table 6). The relationship between EQ-C and the Calculation Skills composite score was not mediated by the Social Communication or the Autistic Mannerisms subscales. These results suggest that the link between empathizing and math achievement may be related to social awareness and cognition rather than autistic behaviors.

### Social Ability as a Potential Mediator between Empathizing and Mathematical Achievement in Boys vs. Girls

**Figure 2:**
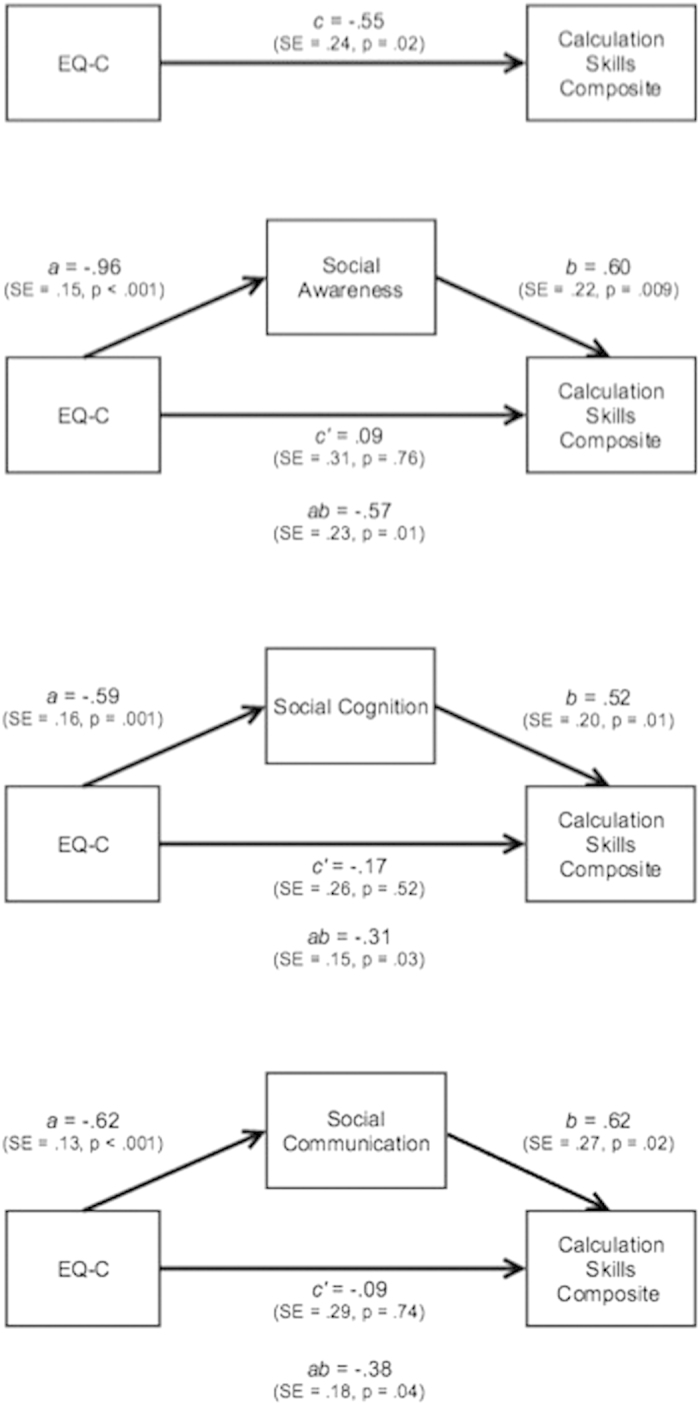
Mediation analyses with SRS measures as mediators between EQ-C and Math Calculation Skills in the whole group, and in boys and girls separately. Bonferroni correction α = 0.0125.

We next evaluated mediation models in each gender in order to assess whether the mediation effects differed by gender. When considering girls alone, there was a significant indirect effect of Social Cognition, Social Awareness and Social Communication in mediating the relationship between EQ-C and the Calculation Skills composite (Fig. 2; Table 6). None of the mediation models were significant in boys (Table 6). There was a significant difference between boys and girls only for the Social Awareness subscale (*t* = −2.36, *p* = 0.02). The relationship between EQ-C and the Calculation Skills composite was not mediated by the Autistic Mannerisms subscale in either boys or girls. These results demonstrate that Social Awareness plays a differential role in mediating the relationship between EQ-C and math achievement in girls.

## Discussion

The central goal of our study was to investigate links between the empathizing and systemizing constructs of the E-S theory and math achievement in children. We examined two dimensions of math achievement – pure symbolic calculation skills (timed and untimed) and contextualized mathematical reasoning – in a sample of TD children. Following research linking systemizing to math skills in autism, we hypothesized that math achievement would be positively related to systemizing. Contrary to our hypothesis, we found no relationship between systemizing and math achievement after controlling for domain general abilities and no relationship between the systemizing brain type (greater discrepancy between systemizing and empathizing) and math achievement. In contrast, math achievement was related to empathizing, with higher EQ-C scores predicting lower scores on the Calculation Skills composite. Further analyses using the Social Responsiveness Scale (SRS)^30^ revealed that the relationship between EQ-C and math achievement was mediated by social ability rather than autistic behaviors. Girls showed stronger empathizing skills than boys, and mediation analyses revealed that social awareness was a strong mediator of the relation between empathizing and math achievement in girls, but not boys. Our findings identify empathizing and social abilities as previously unknown negative predictors of math achievement in school-aged children.

### Systemizing and Mathematical Achievement

Enhanced math achievement has been reported in some individuals with ASD^14^,^33^. Baron-Cohen has argued that the link between ASD and math ability is driven by a tendency toward systemizing, as mathematics can be conceptualized as a rule-based system. Providing a direct link between the E-S theory and mathematics would have implications for a better understanding of a mechanism underlying the connection between ASD and enhanced math abilities, as well as implications for understanding variation in math achievement within the TD population. Despite this theoretical grounding, we found little evidence of a relationship between systemizing and math achievement in our sample. Calculation skills were not correlated with SQ-C. Scores on Applied Problems were marginally positively correlated with SQ-C; however, SQ-C did not significantly explain a portion of the variance in Applied Problems after accounting for FSIQ and reading achievement, known predictors of math achievement.

### Gender Differences in Systemizing and Mathematical Achievement

A marginally significant gender difference on SQ-C was observed in our sample, with girls scoring lower than boys. We detected an effect size of Cohen’s d = 0.39, which is comparable to the effect size of Cohen’s d = 0.40 found by Auyeung and colleagues^13^ in a larger sample of 1,256 participants. Limited statistical power due to the sample size (n = 112) may have played a role in the marginally significant gender differences observed in our study. Based on the effect size reported by Auyeung and colleagues^13^ (d = 0.40), a sample size of approximately 200 would be needed to obtain a statistical power at the 0.80 level. It is possible that our sample size was not large enough to detect this difference and that a larger sample may have yielded a sex difference on SQ-C or a differential relationship between SQ-C and math achievement in boys relative to girls. However, it should also be noted that Auyeung and colleagues’ large pediatric sample^13^ was a convenience sample from an epidemiological school-based study that did not assess IQ measures and other potential confounding influences on SQ-C^13^. In contrast, our sample of 112 children underwent extensive neuropsychological assessments and we controlled for IQ and other potential confounding influences on SQ-C.

### Difference Score, Brain Types, and Mathematical Achievement

EQ-C and SQ-C scores were not correlated, suggesting the constructs are independent of one another. This is in line with Auyeung and colleagues^13^, who did not find a relation between EQ-C and SQ-C in either TD or ASD children, when the groups were examined separately.

In order to further examine empathizing and systemizing in relation to mathematics, we also included analyses using the Difference Score (the discrepancy between EQ-C and SQ-C scores) and by dividing children into ‘brain types’ based on percentile cut offs of the Difference Score^12^. Even if empathizing and systemizing exist as two independent cognitive factors, rather than on one continuum, it is still interesting to consider the consequences of having extreme behavioral tendencies toward one. In line with the Extreme Male Brain theory, we found that males had larger discrepancies between their empathizing and systemizing scores than females did. The discrepancy between systemizing and empathizing did not significantly predict Applied Problems. Although there was a relationship between the Difference score and the Calculation Skills composite, the Difference score did not explain variance in the Calculation Skills composite after accounting for EQ-C, FSIQ, and reading achievement. Dividing participants into ‘brain types’ revealed no significant differences between the groups for either measure of math achievement. It should be noted that the lack of significant differences in math achievement between brain types may be due to the limited sample size.

### Empathizing and Mathematical Achievement

We found a strong relation between EQ-C and the Calculation Skills composite. Specifically, we found a negative relationship with greater empathizing predicting lower calculation ability, which was robust to the inclusion of FSIQ and reading achievement in the model. To further understand the relationship between empathizing and calculation skills, we examined the contributions of math anxiety and social abilities, indexed by the SEMA and SRS, respectively. Consistent with previous research^4^, math anxiety was related to calculation ability but it was not related to empathizing. Instead, we found that social abilities accounted for the relationship between empathizing and math achievement. In particular, measures of social awareness (the ability to pick up social cues), social cognition (the ability to interpret those cues), and social communication (understanding and expression through language) were all correlated with both Calculation Skills and EQ-C in the whole group. Autistic mannerisms did not mediate the relationship between EQ-C and math achievement, suggesting that the connection between empathizing and math achievement is related to social ability rather than an indirect connection to low empathizing resulting from autistic behavioral tendencies.

Taken together, these results suggest that empathizing is a previously unknown independent predictor of math achievement. No previous research has linked empathizing directly to mathematics; however, research by Focquaert and colleagues^34^ found that adults in the STEM disciplines such as engineering and mathematics have a lower tendency to empathize than their peers in the humanities, and that this effect is independent of any gender differences in empathizing.

What potential mechanisms might explain this unexpected relationship? One possibility is that greater empathy may have a distracting effect in the classroom. Attending to the emotional states of individuals in the environment may occupy cognitive resources that would otherwise be devoted to instruction. For instance, research suggests that divided attention and mental ruminations may result in a reduction in working memory level available to solve problems^23^,^35^. Importantly, reading achievement was not related to EQ-C, pointing against a broad effect of empathizing leading to divided attention in the classroom and suggesting instead that sensitivity to emotional states may be particularly detrimental during mathematics instruction. It should be noted that the SRS social abilities subscales were not designed as a measure of the awareness of gender stereotypes, or distractibility in the classroom. Further research that directly assesses these constructs is needed to test these hypotheses.

Thus far, we have framed our findings in terms of the effects of high empathizing on poor math performance. Yet these results equally imply that low empathizing may be beneficial for acquiring math skills. In this view, mathematical strengths may stem, in part, from a lack of distraction by social matters. Perhaps, by not engaging in the social world, children may “free up” time and mental resources for learning mathematical material.

### Gender Differences in Social Ability and Mathematical Achievement

Notably, in our elementary school-aged sample, math achievement did not differ between boys and girls. Although no gender differences in this age group were expected, previous research suggests that precursors to differences in math achievement can be detected in elementary school, including spatial ability^26^,^27^ and strategy use^28^.

Social awareness was a significantly stronger mediator of the relationship between EQ-C and math achievement in girls than in boys. Social awareness, in particular, was the strongest predictor of both math achievement and EQ-C in girls. Based on the mediating role of social awareness in our study, we speculate on a potential mechanism for this relationship. Girls who are more socially aware may be more likely to pick up on societal messages regarding gender roles in math. Analogous to research linking female teachers’ math anxiety to math achievement in girls^1^, our results suggest that higher empathy may impact students who are more tuned to their teacher’s negative emotional state than the content of their lesson. Beilock and colleagues^1^ found that girls who endorsed gender-stereotyped beliefs about math ability had worse math achievement than girls who did not. Thus, young girls who have more social awareness may be more likely to be negatively influenced by the transmission of gender-stereotyped beliefs.

In summary, our findings suggest that greater social awareness in girls may be one of the contributors to the later divergence of math achievement between the genders. Further research is needed to determine the mechanisms underlying the effects of social abilities on math achievement, and in particular whether empathizing or social awareness may be predictors of future declines in math performance.

## Conclusion

The present study is the first to directly examine the relationship between E-S theory and math achievement in TD children. We found no evidence for a relationship between systemizing and math achievement after accounting for general cognitive and reading abilities. There was, however, a negative association between empathizing and calculation ability that was more pronounced in girls. This relationship was mediated by social abilities and not by autistic mannerisms, indicating that skills in picking up social cues may result in poorer math achievement. Social awareness was found to play a differential role in mediating the relationship between EQ-C and math achievement in girls. One interpretation is that the tendency toward social awareness makes girls, but not boys, susceptible to the social transmission of negative gender stereotypes in math. It is particularly interesting that such a differential relationship exists at an early stage of mathematical learning, suggesting that social abilities may also be a predictor of later math achievement. Further research utilizing longitudinal methods is needed to test this hypothesis and investigate the effects of empathizing and systemizing in relation to developmental trajectories of mathematics learning.

## Methods

### Participants

The study protocol was approved by the Stanford University Institutional Review Board. The study was carried out in accordance with relevant guidelines and regulations, and written informed consent from the legal guardian and assent from the child were obtained prior to participation. Families were recruited from the San Francisco Bay Area using mailings to schools and postings in public settings. Flyers targeted children across the math abilities spectrum. Prior to inclusion in the study, parents completed a questionnaire that screened for history of psychiatric illness or medication use. If the child had no history of psychiatric illness or medication use, he or she participated in a neuropsychological assessment session. Intelligence was assessed using the Wechsler Abbreviated Scale of Intelligence (WASI)^31^. Children were excluded if they scored below a standard score of 80 (n = 1) or above a standard score of 140 (n = 4) on the WASI full-scale IQ (FSIQ). Children were also excluded for scoring in the clinical range on the Child Behavior Checklist^36^ syndrome scale (n = 13). The final sample included 112 typically developing children (55 males, 57 females) ages 7 to 12 years (*M* = 8.45, *SD* = 0.98). Males and females did not significantly differ by age (*t*(110) = −1.44, *p* = 0.15). An additional four children (1 male, 3 female) did not have Social Responsiveness Scales, and were excluded from analyses that used this measure.

### Empathizing and Systemizing Measure

The primary guardian of each child completed the Combined Empathy Quotient-Child (EQ-C) and Systemizing Quotient-Child (SQ-C)^13^. The questionnaire was designed to be parent-report in order to avoid variance associated with children’s reading and comprehension abilities. Responses were given using a 4-point Likert scale (*definitely agree, slightly agree, slightly disagree*, and *definitely disagree*). Some items are worded to endorse the trait, while other items are worded to describe low levels of the trait. Items were scored such that two points were given for a strong empathizing or systemizing response and one point was given for a slight empathizing or systemizing response. The empathizing and systemizing items were summed separately to produce an EQ-C and an SQ-C score. Raw EQ-C scores are out of 54 items, while raw SQ-C scores are out of 56 items. The Difference score was computed by subtracting the standard EQ-C score from the standard SQ-C score and dividing by two (see guidelines in Auyeung *et al*.)^13^. Brain types were assigned according to percentiles on the Difference score (see Auyeung *et al*. for percentiles that correspond to each group)^12^,^13^. Due to the small number of participants in the Extreme Types (Extreme Type S: n = 0; Extreme Type E: n = 2) these participants were collapsed into the Type S and Type E groups, respectively.

### Achievement Measures

Participants completed five subtests of the Woodcock Johnson III, Form A^32^. A Basic Reading composite measure was calculated by combining performance on two subsets: Letter Identification, a measure of recognition of visual word forms and knowledge of pronunciations associated with those forms, and Word Attack, a measure of the pronunciation of pseudo words^32^,^37^.

The children also completed the Calculation, Math Fluency, and Applied Problems subtests as measures of math achievement^32^. Calculation is an untimed, paper-and-pencil test designed to measure knowledge of numbers and calculation procedures, including addition, subtraction, multiplication, division, algebra and trigonometry^37^. Grade-appropriate examples include problems such as ‘5 − 1=’ (presented horizontally) and ‘89 − 18=’ (presented vertically). Math Fluency is a measure of speeded application of arithmetic procedures^37^. Problems included a mix of addition, subtraction, and multiplication with operands up to 10. Children were given 3 minutes to complete as many as possible^32^. We computed a Calculation Skills composite measure, which combines the Calculation and Math Fluency subtests, in order to have a single measure capturing arithmetic ability. Applied Problems was also included to assess conceptual understanding through the application of calculation and quantitative reasoning to word problems. These problems included counting, time, money, measurement and multi-step problem solving. Grade-appropriate examples include verbal and visual presentation of the question: “Four people each have six dollars. How much money do they have together? ” or “A foot-long ruler is divided into six equal parts. How long is each part?”^32^

### General Intelligence Measure

Intelligence was assessed using the full-scale intelligence quotient (FSIQ) of the Wechsler Abbreviated Scale of Intelligence (WASI)^31^. The WASI consists of two verbal subtests (Vocabulary and Similarities) and two nonverbal subtests (Block Design and Matrix Reasoning). Performance on all four subtests were converted to age-adjusted standardized scores and used to generate an estimate of FSIQ.

### Math Anxiety Measure

Each child completed the Scale for Early Mathematics Anxiety^4^ with one-on-one guidance from an assessor. The SEMA is designed to assess math anxiety in 2nd and 3rd graders, and was modeled after the Math Anxiety Rating Scale (MARS)^38^ and MARS-E^39^ for early elementary-aged children. The questionnaire has 20 items; half investigate anxiety related to completing problems with mathematical content, and the other half investigate anxiety stemming from situations that require the use of math. The items with mathematical content are based on a content analysis of 2nd and 3rd grade curricula, and include concepts like number sense, basic mathematical functions, measurement, geometry, and mathematical reasoning. For all items, children were asked to rate how anxious the questions made them feel on a 0- to 4- point Likert scale (*not nervous at all, a little nervous, somewhat nervous, very nervous*, and *very very nervous*). Where responses were missing, the mean value of that participant’s other scores was used, and questionnaires with more than 2 items per section missing were excluded. SEMA scores were calculated by summing the points across the 20 items.

### Social Abilities Measure

The child’s primary guardian also completed the Social Responsiveness Scale (SRS)^30^, a quantitative measure of autistic traits. This measure has 65 items, each describing a social behavior or characteristic. Responses take the form of a 0- to 3-point Likert scale (*not true, sometimes true, often true*, and *almost always true*). The child’s guardian was instructed to make responses based on the child’s behavior as presented in the 6 months prior. Scores for all 65 items were summed to produce an SRS Total raw score (maximum score of 195), and mapped to the corresponding T-score. The total score can be broken down into subscales that differentiate types of social ability, including Social Awareness (the ability to pick up on social cues), Social Cognition (the ability to interpret those cues), Social Communication (expressive communication and language interpretation), Social Motivation (the extent to which the child is motivated to engage in interpersonal behaviors), and Autistic Mannerisms (stereotypical behaviors and restricted interests). Where responses to particular items were missing, the median value for that item was used. Items in the SRS that describe prosocial behaviors are reverse scored. Thus, higher scores on the SRS subscales indicate a lack of social ability and more severe autistic mannerisms, and the scale produces an index of psychosocial dysfunction. Scores can range from normal, to mild/moderate, to severe. Severe scores are strongly associated with a clinical diagnosis on the autism spectrum.

### Data Analyses

Pearson correlations were computed to examine the relationship between EQ-C, SQ-C, and two types of math achievement—arithmetic ability as measured by the Calculation Skills composite, and mathematical reasoning skills, as measured by the Applied Problems subtest. The significant relationships were further examined with hierarchical regressions that used SQ-C and EQ-C to predict math achievement after accounting for FSIQ and reading achievement. A stepwise regression was used to test whether there is a unique contribution of the Difference score to the explained variance in math achievement relative to EQ-C, after accounting for FSIQ and reading achievement. ANOVAs were also used to examine whether there are differences between brain types on math achievement, and ANCOVAS were used to examine these differences after accounting for domain general abilities. Mediation analyses were used to examine the role of math anxiety and social abilities in explaining the potential relationship between empathizing and math achievement.

## Additional Information

**How to cite this article**: Escovar, E. *et al*. The Empathizing-Systemizing Theory, Social Abilities, and Mathematical Achievement in Children. *Sci. Rep*. **6**, 23011; doi: 10.1038/srep23011 (2016).

## Supplementary Material

Supplementary Information

## Figures and Tables

**Table 1:** Social and cognitive measures in boys and girls.

Measure	Gender				*t*	*p*
	Boys (N = 55)		Girls (N = 57)			
	M	SD	M	SD		
Age	8.58	1.17	8.32	0.74	−1.44	0.15
FSIQ	112.65	14.64	109.61	12.87	−1.17	0.25
Reading	111.71	9.47	108.51	9.97	−1.74	0.09
Math: Calculation Skills	107.89	16.92	108.72	14.65	−0.61	0.54
Math: Applied Problems	110.45	13.24	105.51	13.98	−1.92	0.06
EQ-C	33.95	7.26	38.79	7.59	3.45	<0.01
SQ-C	23.73	7.69	20.91	7.87	−1.91	0.06

FSIQ = Full-Scale IQ; EQ-C = Empathy Quotient-Child; SQ-C = Systemizing Quotient-Child.

**Table 2:** Relation between SQ-C and cognitive measures.

SQ-C	Boys and Girls (N = 112)		Boys (N = 55)		Girls (N = 57)	
	*r*	*p*	*r*	*p*	*r*	*p*
FSIQ	0.06	0.56	0.10	0.49	−0.03	0.85
Reading	0.16	0.09	0.03	0.85	0.24	0.08
Math: Calculation Skills	0.07	0.44	0.11	0.43	0.02	0.91
Math: Applied Problems	0.16	0.08	0.09	0.53	0.18	0.18

**Table 3:** Relation between EQ-C and cognitive measures.

EQ-C	Boys and Girls (N = 112)		Boys (N = 55)		Girls (N = 57)	
	*r*	*p*	*r*	*p*	*r*	*p*
FSIQ	0.03	0.76	−0.01	0.93	0.15	0.26
Reading	−0.11	0.25	0.02	0.88	−0.14	0.31
Math: Calculation Skills	−0.22*	0.02	−0.14	0.32	−0.29*	0.03
Math: Applied Problems	−0.01	0.91	0.03	0.84	0.07	0.62

^*^Correlation is significant at the 0.05 level (2-tailed).

**Table 4:** Hierarchical regression analysis of Math Calculation Skills.

Calculation Skills	*R*^*2*^	*R*^*2*^ *change*	*β*	SE	*t*	*p*
Model 1	0.23					
FSIQ			0.32	0.11	2.96	0.004
Reading			0.46	0.15	3.08	0.003
Model 2	0.27	0.038				
FSIQ			0.34	0.11	3.23	0.002
Reading			0.41	0.15	2.78	0.006
EQ-C			−0.40	0.17	−2.39	0.019

Model 1: variance accounted for by FSIQ and reading; Model 2: additional variance accounted for by EQ-C after controlling for effects of domain general predictors.

**Table 5:** Relation between Math Calculation Skills and each of the SRS social subscales.

SRS Subscale	Boys and Girls (N = 108)		Boys (N = 54)		Girls (N = 54)	
	Calculation Skills	EQ-C	Calculation Skills	EQ-C	Calculation Skills	EQ-C
Social Awareness	0.26**	−0.51**	0.04	−0.34*	0.44**	−0.67**
Social Cognition	0.30**	−0.49**	0.17	−0.45**	0.42**	−0.45**
Social Communication	0.24*	−0.58**	0.11	−0.52**	0.40**	−0.57**
Social Motivation	0.07	−0.22*	−0.01	−0.28*	0.15	−0.16
Autistic Mannerisms	0.14	−0.25**	−0.05	−0.26*	0.31*	−0.28*

Partial correlations were computed after controlling for FSIQ and reading achievement. The relation between EQ-C and each of the SRS social subscales is also shown.

^*^Correlation is significant at the 0.05 level (2-tailed).

^**^Correlation is significant at the 0.01 level (2-tailed).

**Table 6:** Mediation analysis with SRS measures as mediators between EQ-C and Math Calculation Skills in the whole group, and in boys and girls separately.

SRS Subscale	Boys and Girls (N = 108)		Boys (N = 54)		Girls (N = 54)	
	*z*	*p*	*z*	*p*	*z*	*p*
Social Awareness	−1.66	0.09	0.21	0.83	−2.51*	0.01
Social Cognition	−2.10*	0.03	−0.64	0.52	−2.12*	0.03
Social Communication	−1.36	0.17	−0.04	0.97	−2.09*	0.04
Autistic Mannerisms	−1.13	0.26	0.36	0.72	−1.38	0.17

^*^Correlation is significant at the 0.05 level (2-tailed).
