# Supplementary material for: The Empathizing-Systemizing Theory, Social Abilities, and Mathematical Achievement in Children
Source: Sci Rep. 2016 Mar 14;6:23011. doi: 10.1038/srep23011 (PMC4789644; doi:10.1038/srep23011)
Supplement: Supplementary Information [file srep23011-s1.doc]

**Supplementary Materials**

The Empathizing-Systemizing Theory, Social Abilities and Mathematical Achievement in Children

*Emily Escovar*^1^, Miriam Rosenberg-Lee^1^, Lucina Q. Uddin^2^ and Vinod Menon^1^*

^1^Department of Psychiatry and Behavioral Sciences

Stanford University, Stanford, CA USA

^2^Department of Psychology, University of Miami, Coral Gables, FL USA

*Supplementary Table S1. Relation between age, FSIQ, reading achievement, and measures of math achievement.*

|  | Age | FSIQ | Basic Reading | Calculation  Skills | Applied Problems |
| --- | --- | --- | --- | --- | --- |
| Age | --- | .15 | .03 | .14 | .06 |
| FSIQ |  | --- | .45** | .41** | .64** |
| Reading |  |  | --- | .42** | .57** |
| Calculation Skills |  |  |  | --- | .51** |
| Applied Problems |  |  |  |  | --- |

** Correlation is significant at the .001 level (2-tailed).

*Supplementary Table S2. Hierarchical regression analysis of Math Applied Problems. Model 1: using IQ and reading as domain general predictors; Model 2: additional variance accounted for by SQ-C after controlling for effects of domain general predictors.*

| **Applied Problems** | ***R^2^*** | ***R^2^ change*** | ***β*** | **SE** | ***t*** | ***p*** |
| --- | --- | --- | --- | --- | --- | --- |
| **Model 1** | .50 |  |  |  |  |  |
| FSIQ |  |  | .48 | .08 | 6.29 | < .001 |
| Reading |  |  | .49 | .11 | 4.62 | < .001 |
| **Model 2** | .51 | .01 |  |  |  |  |
| FSIQ |  |  | .48 | .08 | 6.32 | < .001 |
| Reading |  |  | .47 | .11 | 4.39 | < .001 |
| SQ-C |  |  | .14 | .12 | 1.20 | .231 |
